# Independent Multicentre Validation of the ‘Six‐Point’ Model for Malignant Transformation Risk in Oral Epithelial Dysplasia

**DOI:** 10.1111/odi.70173

**Published:** 2025-12-26

**Authors:** Hanya Mahmood, Mike Bradburn, Nadim M. Islam, Omar Kujan, Nasir Rajpoot, Alan Roger Santos‐Silva, Pablo Agustin Vargas, Jacqueline James, Paul Nankivell, Hisham Mehanna, Syed Ali Khurram

**Affiliations:** ^1^ School of Clinical Dentistry, Faculty of Health University of Sheffield Sheffield UK; ^2^ Clinical Trials Research Unit, Sheffield Centre for Health and Related Research University of Sheffield Sheffield UK; ^3^ Department of Oral & Maxillofacial Diagnostic Sciences, College of Dentistry University of Florida Gainesville Florida USA; ^4^ Oral Diagnostic and Surgical Sciences Division, UWA Dental School The University of Western Australia Perth Western Australia Australia; ^5^ Department of Computer Science, Tissue Image Analytics Centre University of Warwick Coventry UK; ^6^ Oral Diagnosis Department, Piracicaba Dental School University of Campinas (UNICAMP) São Paulo Brazil; ^7^ Precision Medicine Centre, Patrick G. Johnston Centre for Cancer Research Queen's University Belfast Belfast UK; ^8^ Institute of Head and Neck Studies and Education, Department of Cancer and Genomic Sciences University of Birmingham Birmingham UK

**Keywords:** histological grading, malignant transformation, oral epithelial dysplasia, outcome prediction, prognosis

## Abstract

**Background:**

Histological grading of oral epithelial dysplasia (OED) is used for predicting malignant transformation risk. However, grading is associated with significant subjectivity and observer variability leading to inconsistency in prognosis prediction. Alternate histological feature‐specific models (‘six‐point’ and ‘two‐point’) have been shown to have potentially better prognostic reliability than conventional grading. This study conducts a multicentre validation of these models.

**Methods:**

102 OED cases (dating 2012–2017) were acquired from 4 independent centres (13 (13%) Sheffield; 40 (39%) Belfast; 30 (29%) Birmingham; 19 (19%) Piracicaba, Brazil) were independently scored using the ‘six‐point’ and ‘two‐point’ models. Feature prevalence, observer agreement and malignant transformation risk were evaluated and compared to conventional grading systems.

**Results:**

The ‘six‐point’ system demonstrated superior predictive value (AUROC of 0.81) compared to the ‘two‐point’ system (AUROC = 0.73, *p* = 0.004), WHO grading (AUROC = 0.71, *p* = 0.03) and binary grading (AUROC = 0.68, *p* = 0.009). Transformation rate for the ‘six‐point’ model was 50% (95% CI 27%–78%) when all 6 features were present compared to 14% (95% CI 5%–32%) when 2–3 features were present.

**Conclusions:**

This study supports the superior performance of the ‘six‐point’ system for transformation prediction on a multicentric sample. Findings indicate that feature‐specific models may be more reliable than existing histological grading systems for prognosis prediction.

**Abbreviations**
AUROC: area under the receiver operating characteristic curve
CI: confidence interval
H&E: haematoxylin and eosin
OED: oral epithelial dysplasia
OSCC: oral squamous cell carcinoma
TRIPOD: transparent reporting of a multivariable prediction model for individual prognosis or diagnosis
WHO: world health organisation
WSI: whole slide imaging

## 1 Background

Oral epithelial dysplasia (OED) is a histopathological condition characterised by epithelial changes in lesions of the oral cavity that have an increased risk of progression to oral squamous cell carcinoma (OSCC) (Pindborg et al. 2012). The development of OED is primarily driven by genomic alterations initiated by chemical carcinogens found in substances such as tobacco, alcohol and areca nut (Tilakaratne et al. 2019). In a smaller proportion of cases, viral infections, particularly human papillomavirus (HPV), can contribute to OED development (de la Cour et al. 2020). Clinically, OED most frequently appears as white, red or mixed lesions, though it can also be seen at the margins of chronic ulcers or within seemingly healthy epithelium. The prevalence of OED varies worldwide, influenced by regional habits and demographic factors. A retrospective cohort study involving 144 OED cases revealed that 42% progressed to OSCC over a 21‐year period (Gómez‐Armayones et al. 2022).

Diagnosing OED is notably complex due to its overlapping features with various inflammatory oral conditions. Histological diagnosis is achieved by identifying a wide range of architectural and cytological characteristics, as outlined in the World Health Organization (WHO) grading system (WHO 2024). However, this grading method oversimplifies the condition's complexity and is associated with significant inter‐ and intra‐rater variability, which compromises its reliability and reproducibility (Kujan et al. 2007; Nankivell et al. 2013; Hankinson et al. 2024). Another limitation of both the WHO and alternative binary grading (Kujan et al. 2006) systems is their failure to assign prognostic value to individual histological features, presenting challenges in predicting malignant transformation risk (Odell et al. 2021). Until recently, limited research has been conducted on specific histological predictors of OED progression. Despite these limitations, the WHO grading system remains the primary method for predicting cancer risk and guiding clinical treatment decisions for patients with OED (Speight 2007).

Our previous work showed a correlation between individual OED histological features and prognosis for the first time (Mahmood et al. 2022). Six histological features (‘bulbous/drop shaped rete pegs’, ‘hyperchromatism’, ‘loss of epithelial cohesion’, ‘loss of stratification’, ‘suprabasal mitoses’, ‘nuclear pleomorphism’) were found to be associated with a greater risk of malignant transformation and OED recurrence (*p* < 0.036) (Mahmood et al. 2022). These features were used to develop the ‘six‐point’ scoring model in which a single point is allocated for the presence of each of these significant features, and the ‘two‐point’ model in which a single point is allocated for each of two features (among the six) that had the best inter‐rater agreement (‘bulbous/drop shaped rete pegs’, ‘loss of epithelial cohesion’). Both models demonstrated significantly better predictive ability (AUROC ≥ 0.774 for malignant transformation and 0.726 for recurrence) than the 2017 WHO (AUROC 0.601 for malignant transformation and 0.624 for recurrence) and the binary grading systems (AUROC 0.665 for malignant transformation and 0.650 for recurrence) (Mahmood et al. 2022).

This study conducts validity testing of the ‘six‐point’ and ‘two‐point’ scoring models on independent OED cases, making direct comparison to the outcomes presented in the previous study by Mahmood et al. (2022). There were three main objectives: first, to independently validate the prognostic ability of the ‘six‐point’ and ‘two‐point’ scoring models for predicting malignant transformation risk of OED; second, to evaluate the inter‐observer variability of the ‘six‐point’ and ‘two‐point’ scoring models compared with the WHO system (2017 version of the 3‐tiered system) and the binary grading systems; third, to analyse the impact of clinical variables on the prognostic abilities of the ‘six‐point’ and ‘two‐point’ scoring models compared to existing clinical grading systems.

## 2 Methods

### 2.1 Validation Dataset

An independent multicentric retrospective sample of 104 histologically confirmed OED cases was acquired in the form of digital whole slide images (WSI) from four different centres, as follows: The validation sample did not include any cases previously reported in Mahmood et al. (2022).

- School of Clinical Dentistry, University of Sheffield, UK (*n* = 13)
- Precision Medicine Centre, Patrick G. Johnston Centre for Cancer Research, Queen's University Belfast, UK (*n* = 40)
- Institute of Head and Neck Studies and Education (InHANSE), University of Birmingham, UK (*n* = 30)
- Piracicaba Dental School, UNICAMP, Brazil (*n* = 19)

Purposive sampling was used to acquire consecutive cases from these centres between 2012 and 2017 (non‐randomised). The sample represents new 4 μm haematoxylin and eosin (H&E) sections of OED, scanned using either a Hamamatsu NanoZoomer 360 (Hamamatsu Photonics, Japan) or an Aperio CS2 (Leica Biosystems, Germany) digital slide scanner at 40× objective power (0.2258 and 0.2520 mpp, respectively). A secure Google Dive folder was set‐up on centrally provisioned University of Sheffield virtual servers for the transfer of images from external sites and once received transferred to the main project repository on the University research storage infrastructure.

Among the Sheffield cohort, in two cases malignant transformation occurred within 3 months of OED diagnosis and due to the difficulty in reliably confirming whether underlying malignancy was already present, these cases were excluded from analysis. The resulting sample therefore comprised 102 OED cases for independent model validation. Further details of the validation cohort are provided in Table S1.

### 2.2 Inclusion and Exclusion Criteria

Prior to the inclusion of cases in this study, they were blindly reviewed by a Consultant Oral and Maxillofacial Pathologist (SAK) to ensure tissue quality and to review the original histological grade. Where necessary, an updated grade using the El‐Naggar et al. (2017) and binary systems were assigned. The inclusion criteria were varying grades of OED with sufficient epithelial tissue for analysis and availability of minimum 3‐year follow‐up data. Cases with incomplete follow‐up records were excluded to minimise bias; therefore, no included cases were lost to follow‐up. Cases demonstrating histological atypia secondary to inflammatory conditions (i.e., *Candida albicans* infection or lichen planus) were excluded. OED lesions positive for the HPV and lesions with verrucous surface morphology were also excluded as they are distinct entities with reportedly different behaviour that could introduce bias into the sample.

### 2.3 Clinical Data Collection

Clinical data collection included patient age, sex, biopsy site, original histological OED grade and the time to transformation in months. As per the previous study by Mahmood et al. (2022), transformation was confirmed as an OED lesion which had progressed to OSCC at the same clinical site within the follow‐up period. Due to difficulty in confirming OED recurrence status from collaborating centres, the authors decided to exclude this outcome measure for the presented study. Data from the four different centres was recorded in a structured proforma using Microsoft Excel (2016) in an anonymised‐linked format.

### 2.4 Reporting Guidelines and Ethics

The TRIPOD (transparent reporting of a multivariable prediction model for individual prognosis or diagnosis) guidelines were used to strengthen the methodology and reporting of this study (Collins et al. 2015) (see Supporting Information). Ethical approval was granted by the West Midlands–Edgbaston Research Ethics Committee (reference: 18/WM/0335) and the research was carried out in compliance with the Helsinki Declaration.

### 2.5 Histological Feature Assessment

Three assessors, including two experienced clinical‐academic Oral and Maxillofacial Pathologists (NI, SAK) and a clinician with extensive expertise and a specialist interest in OED analysis (HaM) conducted independent histological feature examination. The assessors were given access to the WSI dataset via a secure cloud‐based system and were blinded to the original diagnosis and clinical outcomes.

**FIGURE 1:**
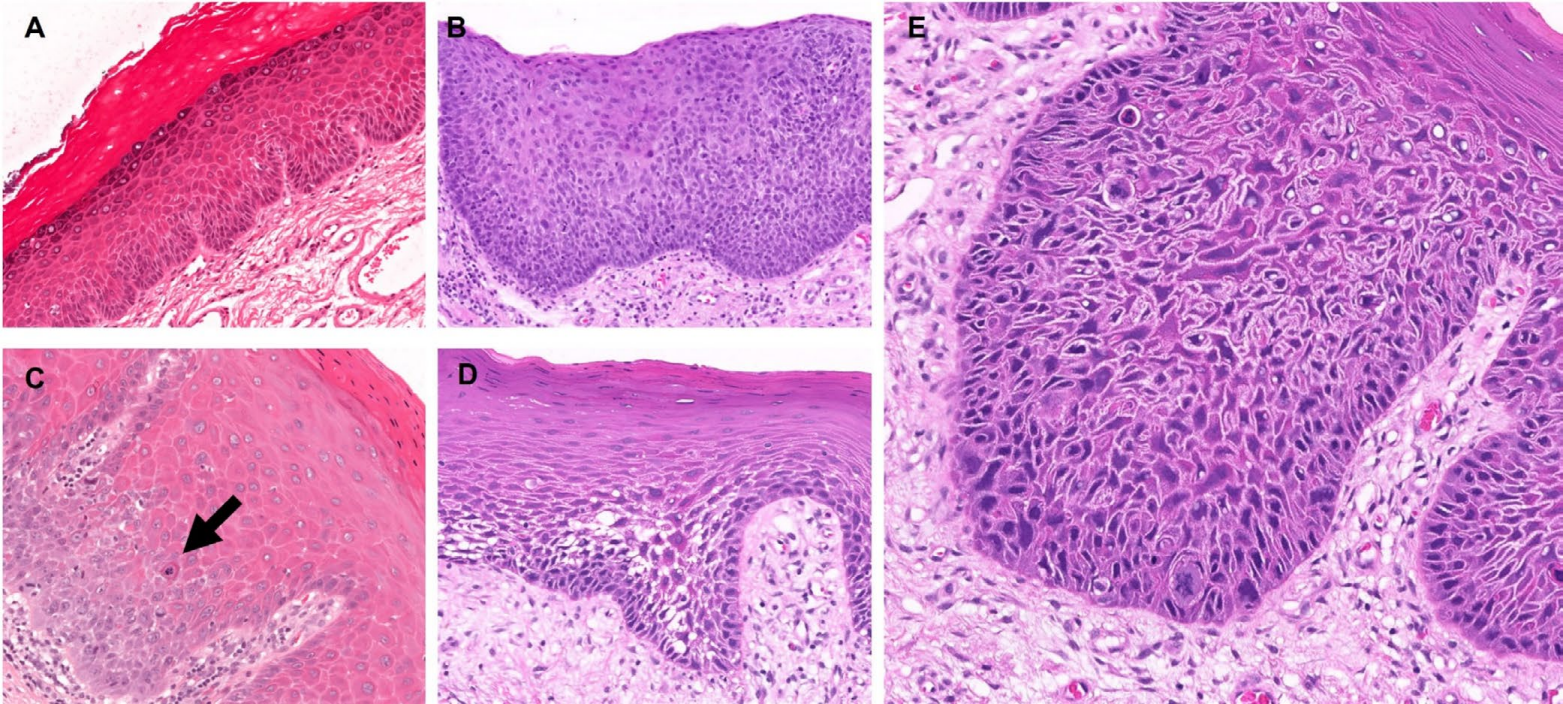
Photomicrographs of H&E‐stained tissue sections (A, B, D—objective ×10; C and E—objective ×20) demonstrating histological features forming the ‘six‐point’ and ‘two‐point’ models. (A) bulbous rete pegs; (B) loss of stratification; (C) suprabasal mitosis (highlighted by black arrow); (D) loss of epithelial cohesion; (E) hyperchromatism and nuclear pleomorphism.

The assessors independently scored the six histological features identified in the previous study by Mahmood et al. (2022) as having adequate inter‐rater agreement and being associated with malignant transformation risk (Figure 1). These six features are a subset of the original 12 histological features evaluated in the previous work: (i) bulbous rete pegs; (ii) hyperchromatism; (iii) loss of epithelial cohesion; (iv) loss of stratification; (v) nuclear pleomorphism; (vi) suprabasal mitosis.

Feature scoring was conducted using a similar approach to the initial study (Mahmood et al. 2022), whereby the assessors were asked to provide a binary score to record the presence or absence of individual features. A score of one was given if the feature was abundantly visible, and a score of zero was given if the feature was absent or rare/focal. Scores were entered into a pre‐developed Microsoft Excel (2016) spreadsheet. Consensus was defined as agreement between at least two of the three raters. We acknowledge that this approach may overestimate reproducibility compared with routine single‐pathologist practice. However, it is common practice for complex OED cases to receive a second opinion for a ‘consensus’ diagnosis and grading.

### 2.6 Statistical Analysis

Statistical analyses were conducted using the Stata Statistical Software (Version 17, 2021) (StataCorp, LLC 2021). All tests were two‐tailed and *p* < 0.05 were considered statistically significant. Three outcomes were measured:

1. Histological feature prevalence

The prevalence of a feature was calculated separately for each assessor, and by ‘consensus’ which was defined as the number of patients for whom at least two assessors considered a feature ‘present’.

2 Observer agreement

Agreement was summarised as the percentage of patients for whom all three raters agreed, and by two chance‐corrected measures (Cohen's kappa coefficient and Gwet's AC1, along with their 95% confidence intervals (CIs)). For the latter two measures, a value of one denotes perfect agreement whilst zero relates to no agreement beyond chance alone. The kappa scores were interpreted based on historical standards, with scores ≤ 0.2 representing ‘slight’, 0.2–0.4 ‘fair’, 0.4–06 ‘moderate’, 0.6–0.8 ‘substantial’ and 0.8–1.0 ‘near perfect’ agreement (Landis and Koch 1977). Whilst the kappa statistic is commonly used, it can be affected by prevalence, therefore the alternative Gwet AC1 measure was also used in this study (Wongpakaran et al. 2013).

3 Malignant transformation

Malignant transformation was analysed using two approaches. The first approach measured the incidence of transformation using the area under the receiver‐operator characteristic (AUROC) curve and its 95% CI to assess predictive ability. The second incorporated the follow up duration using Kaplan Meier to visualise associations between individual pathological features and time to transformation and used Cox regression to calculate Hazard Ratios with 95% CIs.

## 3 Results

### 3.1 Characteristics of the Validation Sample

One hundred and two cases, each from a different patient, were used for independent model validation and analysis. The sample included 40 (39%) cases from Belfast, 30 (29%) from Birmingham, 19 (19%) from Brazil and 13 (13%) from Sheffield. Overall, 28 (27%) were graded as mild OED, 41 (40%) as moderate OED and 33 (32%) as severe OED. Binary grading confirmed 37 (36%) as low grade and 65 (64%) as high‐grade lesions. There was a slightly higher proportion of females (55, 54%) compared to males (47, 46%) with an average age of 58.9 years (IQR 12.7) (Table S1). The median time to malignant transformation was 60 months (IQR 35) with a minimum follow‐up period of 36 months. No included cases were lost to follow‐up.

### 3.2 Feature Prevalence

With the exception of suprabasal mitoses, all six histological features were notably more prevalent in this study compared with the previous study which proposed the two new models (Mahmood et al. 2022). The most prevalent features in this study were ‘hyperchromatism’ (97%) and ‘nuclear pleomorphism’ (90%) and the least prevalent features were ‘loss of epithelial cohesion’ (45%) and ‘suprabasal mitoses’ (24%) (Table 1).

**TABLE 1:** Feature‐specific prevalence^a^ of 102 cases of oral epithelial dysplasia compared with 109 cases reported by Mahmood et al. (2022).

	Current study				
	Consensus^b^	Assessor 1	Assessor 2	Assessor 3	Mahmood et al. (2022)
Bulbous rete pegs	72 (71%)	68 (67%)	67 (66%)	85 (83%)	57%
Hyperchromatism	99 (97%)	97 (95%)	98 (96%)	97 (95%)	54%
Loss of epithelial cohesion	46 (45%)	49 (48%)	28 (27%)	73 (72%)	30%
Loss of stratification	63 (62%)	70 (69%)	61 (60%)	46 (45%)	42%
Nuclear pleomorphism	92 (90%)	94 (92%)	92 (90%)	80 (78%)	36%
Suprabasal mitoses	24 (24%)	23 (23%)	21 (21%)	93 (91%)	45%

^a^ Feature prevalence presented as consensus (defined as two or more assessors recording a positive assessment and separately for each rater).

^b^ Denominator is *N* = 306, or 102 patient samples × three raters.

### 3.3 Observer Agreement

Interobserver agreement was typically modest for all six histological features, although the agreement of certain features differed notably when compared to the previous study by Mahmood et al. (2022) (Table 2). Two features demonstrated greater interobserver variability in this study, namely ‘loss of epithelial cohesion’ (Gwet's AC1 0.29) and ‘suprabasal mitoses’ (Gwet's AC1 0.06).

**TABLE 2:** Interobserver agreement (with 95% CI) with comparison to previous study.

	Complete agreement		Cohen's kappa (95% CI)		Gwet's AC1 (95% CI)	
	Current study	Mahmood et al. (2022)	Current study	Mahmood et al. (2022)	Current study	Mahmood et al. (2022)
Bulbous rete pegs	70 (69%)	72 (66%)	0.49 (0.36, 0.62)	0.54	0.65 (0.53, 0.77)	0.56
Hyperchromatism	93 (91%)	53 (49%)	0.33 (0.01, 0.64)	0.32	0.94 (0.89, 0.98)	0.32
Loss of epithelial cohesion	48 (47%)	80 (73%)	0.34 (0.23, 0.45)	0.58	0.29 (0.16, 0.43)	0.69
Loss of stratification	64 (63%)	61 (56%)	0.50 (0.38, 0.62)	0.41	0.52 (0.39, 0.64)	0.43
Nuclear pleomorphism	70 (75%)	62 (57%)	0.26 (0.10, 0.42)	0.38	0.78 (0.69, 0.87)	0.47
Suprabasal mitoses	29 (28%)	54 (50%)	0.21 (0.13, 0.28)	0.34	0.06 (−0.05, 0.16)	0.34

### 3.4 Malignant Transformation Incidence and Prediction

The incidence of malignant transformation (based on the ‘consensus’ definition) demonstrated that ‘nuclear pleomorphism’ and ‘hyperchromatism’ were commonly recorded both among transformed and non‐transformed cases with little distinction between the two. But the remaining four features, whilst common, demonstrated more apparent separation between those that did and did not transform (Table S2).

As per the previous study, the six studied histological features were used to develop two scoring systems: a ‘six‐point’ system in which each feature is given a single point if present, and a more simplified ‘two‐point’ system based on the two features originally identified as having the best rater agreement (‘loss of epithelial cohesion’ and ‘bulbous rete pegs’). It should be noted that, in this study, ‘loss of epithelial cohesion’ was among the least well‐agreed features.

**FIGURE 2:**
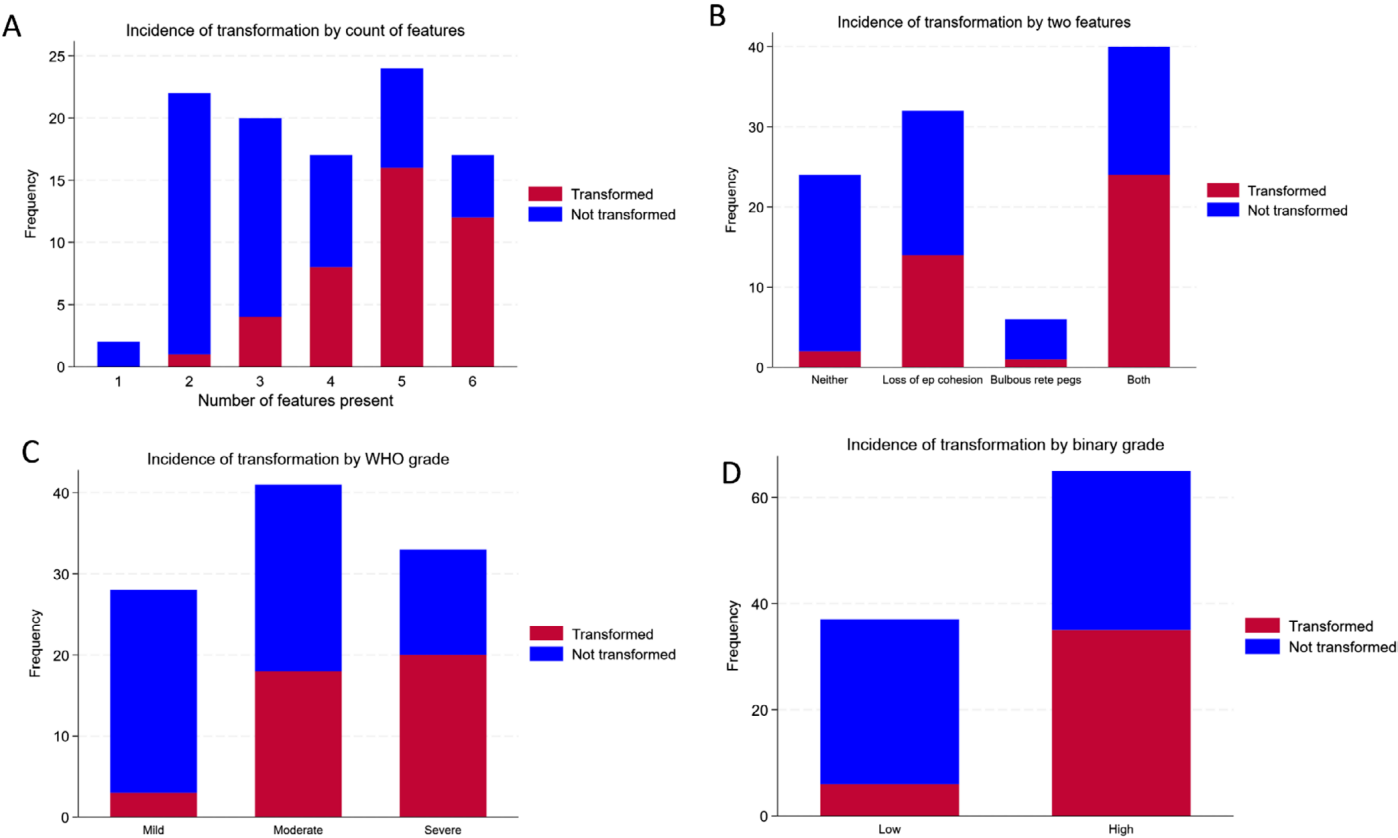
Malignant transformation incidence in relation to scoring approaches. (A) Incidence by count of features using six‐point system; (B) Incidence by count of features using two‐point system; (C) Incidence by WHO grading; (D) Incidence by binary grading. Bar key: Blue = Not transformed, Red = Transformed.

The incidence of malignant transformation in relation to each score is displayed in Figure 2, with comparison to the WHO and binary grading systems. Whilst differences were apparent with both, the most immediately obvious was the ‘six‐point’ system which had an increasing proportion of cases that transformed as the score increased. For the ‘two‐point’ model, the presence of both features (‘loss of epithelial cohesion’ and ‘bulbous rete pegs’) was associated with a higher incidence of malignant transformation than when each of these features was present in isolation.

**FIGURE 3:**
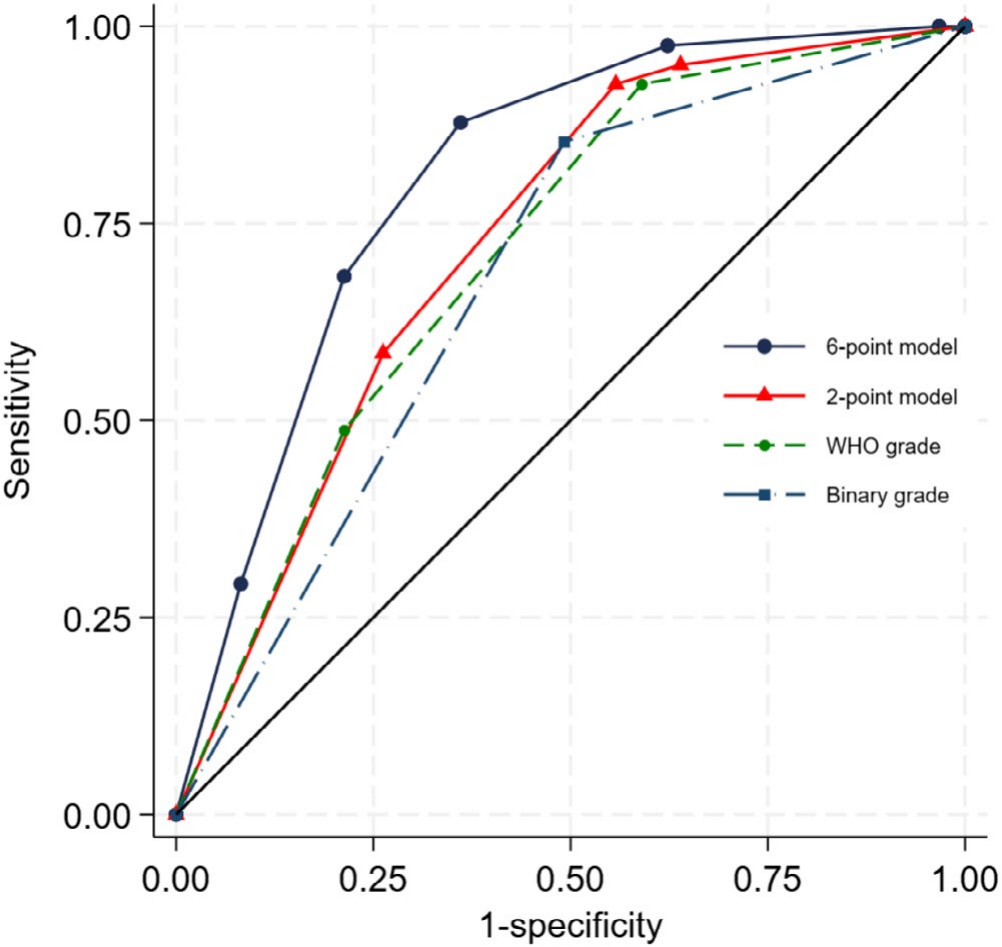
AUROC for malignant transformation in relation to scoring approaches. All four systems showed significantly better than chance discrimination (*p* < 0.0001), but with varying degrees of prognostic strength. The ‘six‐point’ system demonstrated the highest among these with an AUROC of 0.81 and was statistically higher than the ‘two‐point’ system (AUROC = 0.73, *p* = 0.004), WHO grading system (AUROC = 0.71, *p* = 0.03) and binary grading system (AUROC = 0.68, *p* = 0.009). Line Key: Navy blue = six‐point model, Red = two‐point model, Green = WHO grading, Pale Blue = Binary grading.

The AUROC curves for the ‘six‐point’ and ‘two‐point’ scoring approaches compared with the WHO and binary grading systems is presented in Figure 3. All four systems showed significantly better than chance discrimination (*p* < 0.0001), but with varying degrees of prognostic strength. The ‘six‐point’ system demonstrated the highest among these with an AUROC of 0.81 and was statistically significantly higher than the ‘two‐point’ system (AUROC = 0.73, *p* = 0.004), WHO grading system (AUROC = 0.71, *p* = 0.03), and binary grading system (AUROC = 0.68, *p* = 0.009). On univariate analyses, ‘bulbous rete pegs’ alone had an AUROC of 0.68, with ‘loss of stratification’ (AUROC 0.70) and ‘suprabasal mitosis’ (AUROC 0.69) being similar.

### 3.5 Kaplan Meier Analysis for Time to Transformation

**FIGURE 4:**
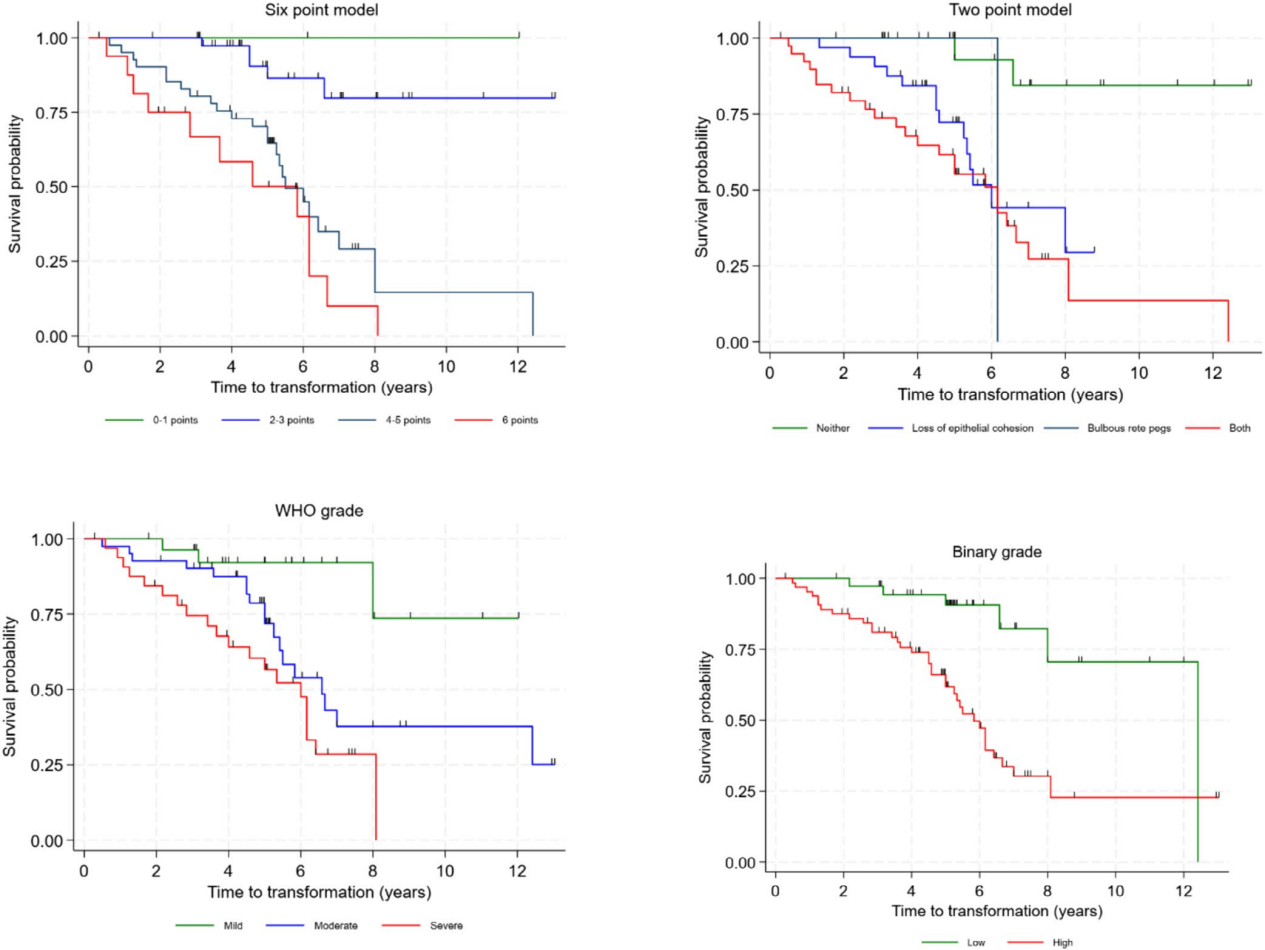
Kaplan Meier curves for time to transformation for different scoring approaches. An estimated 28% (95% CI 20% to 39%) had transformed within 5 years. For the ‘six‐point’ model, the predicted transformation rate when all 6 features were present was 50% (95% CI 27% to 78%) compared to 35% (95% CI 23% to 53%) when 4–5 features were present and 14% (95% CI 5% to 32%) when 2–3 features were present. No transformations were recorded among patients with fewer than 2 features present.

Figure 4 shows the Kaplan Meier survival curves for time to malignant transformation for each of the scoring approaches, in which the curves maintain the separation. Overall, an estimated 28% (95% CI 20% to 39%) had transformed within 5 years. For the ‘six‐point’ model, the predicted transformation rate when all six features were present was 50% (95% CI 27% to 78%) compared to 35% (95% CI 23% to 53%) when 4–5 features were present and 14% (95% CI 5% to 32%) when 2–3 features were present. No transformations were recorded among patients with fewer than two features present.

### 3.6 Effect of Additional Variables on Prognostic Models

The models were also fitted with additional variables (age, sex, histological grading) to see if any combination could improve predictive ability as characterised by the AUROC (Table 3). The ‘six‐point’ model retained the best predictive ability compared to other models, with minor improvements when combined with other histological grading systems and clinical variables. The ‘two‐point’ model along with the WHO and binary grading systems demonstrated modest improvements when combined with other clinical variables.

**TABLE 3:** AUROC (with 95% CI) by scoring system with inclusion of additional variables.

Models	AUROC (95% CI)
6‐point model	0.807 (0.724, 0.889)
6‐point + age + sex	0.814 (0.731, 0.897)
6‐point + WHO grade	0.813 (0.731, 0.895)
6‐point + binary grade	0.820 (0.738, 0.903)
2‐point model	0.729 (0.638, 0.819)
2‐point + age + sex	0.742 (0.647, 0.837)
2‐point + WHO grade	0.763 (0.674, 0.852)
2‐point + binary grade	0.761 (0.672, 0.850)
WHO grade	0.714 (0.621, 0.806)
WHO grade + age + sex	0.740 (0.645, 0.836)
Binary grade	0.681 (0.597, 0.765)
Binary grade + age + sex	0.728 (0.629, 0.826)

Figure S1 demonstrates AUROC and 95% confidence intervals for the different scoring approaches with comparisons between the presented study and the previous one by Mahmood et al. (2022).

### 3.7 Univariate Associations of Individual Features

Based on the assumption that each of the six histological features carry the same weight, the hazard ratios for the individual features are presented in Figure S2. Overall, the hazard ratios from the present study are similar to those observed in the previous study Mahmood et al. (2022) but do suggest that different features have different levels of association with malignant transformation risk. With regard to ‘nuclear pleomorphism’, since most cases had this feature present (92/102) and none of the remaining 10 cases transformed, it was not possible to reliably estimate a hazard ratio.

### 3.8 Prognostic Performance by Assessors

Similar to the previous study, the prognostic performance of the ‘six‐point’ and ‘two‐point’ models was calculated separately for each of the three raters, reflecting how the models are likely to be used in diagnostic practice (Table S3). The AUROC was almost identical for two of the three assessors, whilst the AUROC for the third rater was slightly lower. Overall, the ‘six‐point’ model demonstrated better prognostic ability for the individual raters than the ‘two‐point’ model (AUROC 0.81 vs. AUROC 0.73, respectively).

## 4 Discussion

This study conducts independent validity testing of the previously proposed ‘six‐point’ and ‘two‐point’ scoring models for malignant transformation prediction in OED. Findings demonstrate the ‘six‐point’ model as being the most predictive system (AUROC 0.81) compared to the ‘two‐point’ model, WHO and binary grading systems (AUROC 0.68–73). The ‘two‐point’ model did also perform better than the WHO and binary grading systems, but to a lesser degree (AUROC 0.73). The addition of other variables (Table 3) further improved model performance for both the ‘two’‐ and ‘six‐point’ models, albeit marginally.

A key aspect of histological grading is to assist clinicians with managing individual patients with OED. However, it is evident from this study that existing grading systems are less reliable at predicting prognosis, and there is a need for improvement. Currently, diagnosis and grading of OED using the ‘gold standard’ WHO (2024) classification relies on identification of a wide range of histological architectural and cytological features (28 in total) but this approach places little value on the strength of individual features. This has been further reinforced by a recent study which concluded that variations in the prognostic strength of individual histological features and the use of clinical information may contribute to interobserver variability (Ng et al. 2024). The ‘six‐point’ model was first developed using features identified as having adequate inter‐rater agreement and a statistical association with clinical outcomes (Mahmood et al. 2022). This model demonstrates that assessment of just six histological features with attributed strength can significantly improve predictive ability, and may be more prognostically reliable than identification of a wider range of features. Of note, two of the six features forming this model (‘drop‐shaped rete ridges’ and ‘suprabasal/superficial mitosis’) have been supported in a recent study as the most easily recognised architectural features supporting their relevance and inclusion in the formulation of future OED classifications (Ng et al. 2024).

However, when using the ‘six‐point’ model, the allocation of a single point per feature implies that the six features each have the same importance, a choice that was made pragmatically on two grounds: to avoid overfitting to the data and for ease of use of the eventual score. The assumption of equal weighting was examined by evaluating the hazard ratio of each feature individually (Figure S2), demonstrating that different features have different levels of association with malignant transformation. Therefore, any future improvements of this scoring model may consider giving different weights to the individual features or even removing ‘redundant’ features entirely from the model. Re‐fitting the model to allow the different weights for these features produced an AUROC of 0.82, compared to the 0.81 observed from allocating equal weight to the six individual features.

Interrater reproducibility is a potentially important feature of grading. However, reproducibility does not necessarily reflect accuracy, because it is possible to be reproducibly incorrect. In the present study, despite the considerable interobserver variability for certain histological features assessed individually (i.e., ‘loss of epithelial cohesion’—Gwet's AC1 0.29; ‘suprabasal mitosis’—Gwet's AC1 0.06) (Table 2), when combined, the ‘six‐point’ model still retained good prognostic strength, further supporting its robustness and credibility as a prognostic tool. We emphasise, however, that subjectivity cannot be fully eliminated in histopathological assessment, and the proposed models attempt to reduce, but not eliminate, this variability. This highlights the need for future integration of digital image analysis and AI‐based feature quantification to enhance reproducibility.

Another point of consideration for the newly proposed models is that they are based on the consensus of three raters; for instance, ‘bulbous rete pegs’ is defined as present if at least two of the three assessors considered it present and otherwise absent. This may well overestimate how well the model works in practice where an assessment is made by a single pathologist; introducing measurement error will typically lessen associations. To address this, the ‘two’‐ and ‘six‐point’ models were calculated separately for each assessor (Table S3) and findings have shown that the ‘six‐point’ model has better prognostic ability for the individual raters compared with the ‘two‐point’ model.

The addition of other variables (age, sex, histological grading using conventional systems) only marginally improved model performance (Table 3), supporting findings from the previous study by Mahmood et al. (2022). Due to the retrospective nature of the study, it was not possible to obtain consistent intra‐oral site or social history (smoking, tobacco use, alcohol consumption) information. This limits the assessment of the full clinical applicability of the models. Prospective datasets that integrate these covariates, alongside lesion appearance (i.e., leukoplakia, erythroplakia), will be essential to further refine the predictive models and increase their clinical utility. Future work may also explore how these models may be adapted or applied to other histological conditions that mimic OED, such as HPV‐related OED.

A further limitation is the lack of standardisation in biopsy size, number and site across centres. This may introduce operator‐dependent variability which can influence transformation risk estimates and diagnostic reproducibility. We note that this limitation is inherent to most retrospective studies of OED and emphasises the need for prospective multicentre validation with standardised sampling protocols and randomised case inclusion to provide a more robust platform to validate and refine the proposed models. While this study provides independent multicentre validation, the retrospective design limits generalisability compared with prospective validation.

This study highlights the potential usefulness of using digital WSI for grading and inter‐observer evaluation of OED. In comparison to traditional slide analysis using light microscopy, digital analysis has several advantages including improved accessibility for remote reporting as well as the potential to integrate AI‐based tools for quantitative feature‐based analysis, automated scoring and risk stratification (Shephard, Mahmood, Raza, Khurram, and Rajpoot 2024; Shephard, Mahmood, Raza, Damaceno Araujo, et al. 2024; Shephard, Bashir, Mahmood, Jahanifar, et al. 2024). Other benefits include improved efficiency for slide archiving and storage and broader applications for education and training.

In conclusion, this study independently validates the prognostic ability of the ‘six‐point’ model for OED progression on a multicentric sample of cases. Our findings highlight that the presence of a smaller number of features with high prognostic strength may be potentially more important than the presence of a larger number of features of low to moderate prognostic strength. While the six features forming the ‘six‐point’ model are listed features in the WHO classification, the innovation lies in quantitatively validating their prognostic strength and combining them into a reproducible feature‐specific model. Previous WHO and binary grading systems consider a broad set of features without weighting or evidence of individual prognostic value. By contrast, the proposed ‘six‐point’ model focuses on a reduced subset of features statistically linked to malignant transformation, thereby simplifying and strengthening prognostic prediction. This work provides an evidence‐based refinement of existing histological criteria, offering a pragmatic step toward greater objectivity while paving the way for integration with digital/AI methods in future studies. It is hoped that this approach will provide pathologists, clinicians and patients with more reliable and objective information about the behaviour and progression of OED lesions to guide treatment decisions. Based on the findings from this study, the authors encourage the integration of the ‘six‐point’ model alongside existing grading systems. Meanwhile, a larger scale multi‐centre prospective study to further refine and test the models is required prior to establishing its formal use in clinical practice.

## Author Contributions

**Hanya Mahmood:** conceptualization, investigation, funding acquisition, writing – original draft, methodology, validation, visualization, writing – review and editing, software, formal analysis, project administration, data curation, resources. **Mike Bradburn:** writing – original draft, methodology, validation, writing – review and editing, formal analysis, visualization, resources. **Nadim M. Islam:** writing – review and editing, methodology, investigation, validation, data curation. **Omar Kujan:** investigation, methodology, validation, writing – review and editing, data curation. **Nasir Rajpoot:** investigation, methodology, validation, writing – review and editing, supervision. **Alan Roger Santos‐Silva:** investigation, methodology, writing – review and editing, validation, data curation. **Pablo Agustin Vargas:** investigation, methodology, validation, writing – review and editing, data curation. **Jacqueline James:** investigation, methodology, validation, writing – review and editing, data curation. **Paul Nankivell:** investigation, methodology, validation, writing – review and editing, data curation. **Hisham Mehanna:** investigation, methodology, validation, writing – review and editing, data curation. **Syed Ali Khurram:** conceptualization, investigation, methodology, validation, visualization, writing – review and editing, data curation, supervision, resources, project administration, formal analysis, software, writing – original draft.

## Funding

Ha.M. received funding from the National Institute for Health Research for this study, as part of a Doctoral Research Fellowship (NIHR300904). N.R. and S.A.K. acknowledge funding from Cancer Research UK (C63489/A29674). Hi.M. reports grants from UK National Institute of Health research, Cancer Research UK, the UK Medical Research Council and AstraZeneca; advisory board fees from AstraZeneca, MSD, Merck, Nanobiotix and Seagen; and is Director of Warwickshire Head Neck clinic and Docpsert Health. He is also an NIHR Senior Investigator. The views expressed in this article are those of the author(s) and not necessarily those of the NIHR, or the Department of Health and Social Care.

## Ethics Statement

Ethical approval was granted by the West Midlands–Edgbaston Research Ethics Committee (reference: 18/WM/0335). The study was performed in accordance with the Declaration of Helsinki.

## Conflicts of Interest

N.R. is the co‐founder, Director and shareholder of Histofy Ltd. S.A.K. is a shareholder of Histofy Ltd. The remaining authors declare no conflicts of interest.

## Supporting information


**Data S1:** TRIPOD checklist: Prediction model development.

**Figure S1:** Comparison of malignant transformation prediction between studies.

**Figure S2:** Univariate association between time to transformation and individual features. Since the majority of cases (92/102) had nuclear pleomorphism, and none of the remaining 10 transformed, the hazard ratio could not be reliably estimated.

**Table S1:** Characteristics of the 102 oral epithelial dysplasia cases.

**Table S2:** Malignant transformation incidence in relation to individual histological features.

**Table S3:** Prognostic performance by individual assessor and overall.

## Data Availability

All data generated or analysed during this study are included in this published article and its Supporting Information.
