# Supplementary figures and images for: Independent Multicentre Validation of the ‘Six‐Point’ Model for Malignant Transformation Risk in Oral Epithelial Dysplasia
Source: Oral Dis. 2025 Dec 26;32(5):1273–82. doi: 10.1111/odi.70173 (PMC13365013; doi:10.1111/odi.70173)

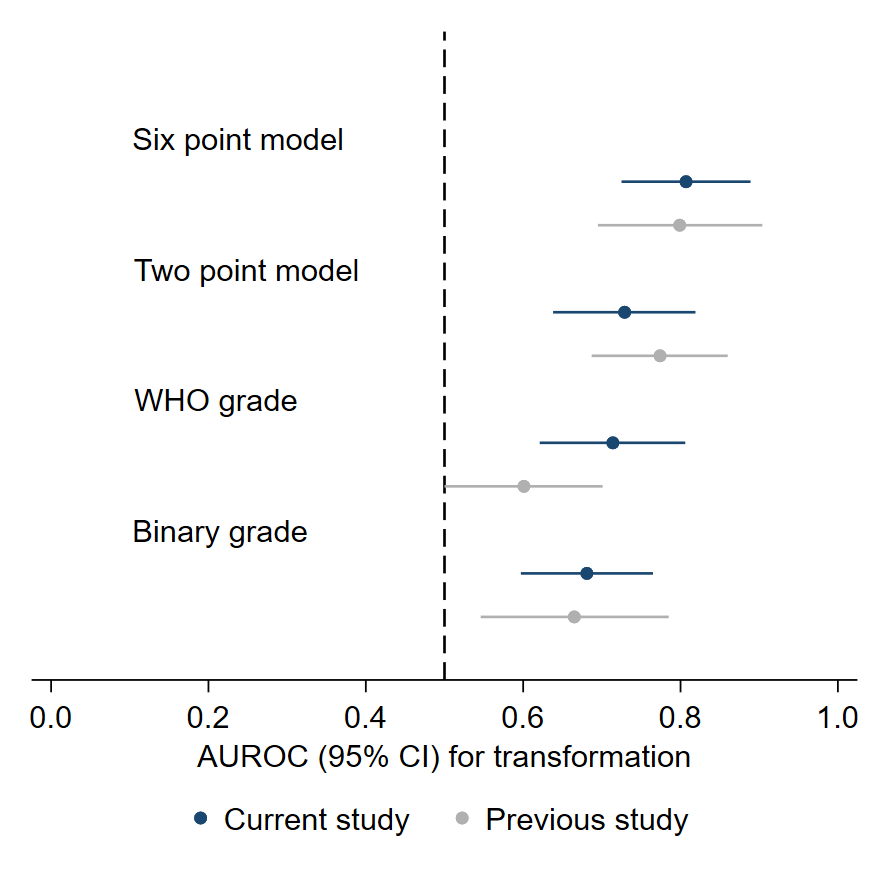


**Supplementary Figure 1.** Comparison of malignant transformation prediction between studies.

Supplement: Supplementary file 2 — Figure S1: Comparison of malignant transformation prediction between studies. [file ODI-32-1273-s004.docx]
