# Supplementary material for: Independent Multicentre Validation of the ‘Six‐Point’ Model for Malignant Transformation Risk in Oral Epithelial Dysplasia
Source: Oral Dis. 2025 Dec 26;32(5):1273–82. doi: 10.1111/odi.70173 (PMC13365013; doi:10.1111/odi.70173)
Supplement: Supplementary file 3 — Figure S2: Univariate association between time to transformation and individual features. Since the majority of cases (92/102) had nuclear pleomorphism, and none of the remaining 10 transformed, the hazard ratio could not be reliably estimated. [file ODI-32-1273-s003.docx]

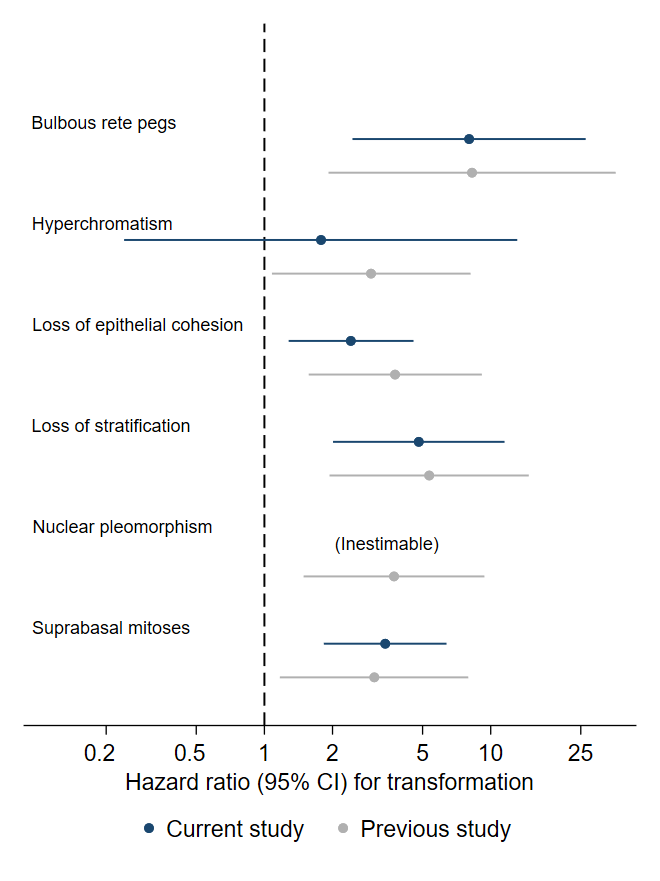


**Supplementary Figure 2.** Univariate association between time to transformation and individual features. Since the majority of cases (92/102) had nuclear pleomorphism, and none of the remaining 10 transformed, the hazard ratio could not be reliably estimated.
