# Supplementary material for: Independent Multicentre Validation of the ‘Six‐Point’ Model for Malignant Transformation Risk in Oral Epithelial Dysplasia
Source: Oral Dis. 2025 Dec 26;32(5):1273–82. doi: 10.1111/odi.70173 (PMC13365013; doi:10.1111/odi.70173)
Supplement: Supplementary file 4 — Table S1: Characteristics of the 102 oral epithelial dysplasia cases. [file ODI-32-1273-s005.docx]

| **Feature** | **N (%) or mean [SD]** |
| --- | --- |
|  |  |
| *Centre* |  |
| Belfast | 40 (39%) |
| Birmingham | 30 (29%) |
| Brazil | 19 (19%) |
| Sheffield | 13 (13%) |
|  |  |
| *Age* | 58.9 [12.7] |
|  |  |
| *Sex* |  |
| Female | 55 (54%) |
| Male | 47 (46%) |
|  |  |
| *Grade (WHO)* |  |
| Mild | 28 (27%) |
| Moderate | 41 (40%) |
| Severe | 33 (32%) |
|  |  |
| *Grade (binary)* |  |
| Low | 37 (36%) |
| High | 65 (64%) |

**Supplementary Table 1.** Characteristics of the 102 oral epithelial dysplasia cases
