# Supplementary material for: Independent Multicentre Validation of the ‘Six‐Point’ Model for Malignant Transformation Risk in Oral Epithelial Dysplasia
Source: Oral Dis. 2025 Dec 26;32(5):1273–82. doi: 10.1111/odi.70173 (PMC13365013; doi:10.1111/odi.70173)
Supplement: Supplementary file 5 — Table S2: Malignant transformation incidence in relation to individual histological features. [file ODI-32-1273-s006.docx]

**Supplementary Table 2.** Malignant transformation incidence in relation to individual histological features.

| **Feature** | **Transformed (N=41)** | **Not transformed (N=61)** |
| --- | --- | --- |
| Bulbous rete pegs | 38 (93%) | 34 (56%) |
| Hyperchromatism | 40 (98%) | 59 (97%) |
| Loss of epithelial cohesion | 25 (61%) | 21 (34%) |
| Loss of stratification | 35 (85%) | 28 (46%) |
| Nuclear pleomorphism | 41 (100%) | 51 (84%) |
| Suprabasal mitoses | 19 (46%) | 5 (8%) |
