# Supplementary material for: Independent Multicentre Validation of the ‘Six‐Point’ Model for Malignant Transformation Risk in Oral Epithelial Dysplasia
Source: Oral Dis. 2025 Dec 26;32(5):1273–82. doi: 10.1111/odi.70173 (PMC13365013; doi:10.1111/odi.70173)
Supplement: Supplementary file 6 — Table S3: Prognostic performance by individual assessor and overall. [file ODI-32-1273-s002.docx]

**Supplementary Table 3.** Prognostic performance by individual assessor and overall.

|  | **Overall** | **Assessor 1** | **Assessor 2** | **Assessor 3** |
| --- | --- | --- | --- | --- |
| 6-point model | 0.81 | 0.81 | 0.80 | 0.75 |
| 2-point model | 0.73 | 0.72 | 0.72 | 0.70 |
